# CROSS-SECTIONAL ASSOCIATION OF FALLS AND POST-TRAUMATIC STRESS IN CANADIANS ACROSS LEVELS OF FRAILTY

**DOI:** 10.1093/geroni/igz038.067

**Published:** 2019-11-08

**Authors:** Olga Theou, Mario Ulises Pérez-Zepeda, Joshua Armstrong, Judith Godin, Melissa Andrew, Susan Kirkland, Kenneth Rockwood

**Affiliations:** 1 Dalhousie University, Halifax, Nova Scotia, Canada; 2 Instituto Nacional de Geriatria, Ciudad de Mexico, Mexico; 3 Lakehead University, Thunder Bay, Ontario, Canada; 4 Division of Geriatric Medicine, Dalhousie University & Nova Scotia Health Authority, Halifax, Nova Scotia, Canada

## Abstract

Frail older adults are vulnerable to stressors and are more likely to experience adverse outcomes. Post-traumatic stress is common in older adults, and can be related to common adverse outcomes, such as falls. We examined whether falls are associated with post-traumatic stress in middle-aged and older Canadians, by levels of frailty. We conducted cross-sectional analysis of the baseline assessment of the Canadian Longitudinal Study on Aging’s tracking cohort, comprising 21,241 individuals, aged 45 to 85 years. We constructed a 60-item frailty index (FI) and defined post-traumatic stress using the primary care post-traumatic stress disorder four-item tool (score 3 as the cut-point). Logistic regressions with post-traumatic stress as the dependent variable and at least one fall in the past year as the independent variable, were adjusted for socio-demographic variables and stratified according to FI 0.1 groups. Prevalence of post-traumatic stress and falls was of 6.5% and 5.0%, respectively for the whole sample. Among those who did not fall prevalence of post-traumatic stress ranged across frailty levels from 3.2% (FI<0.1) to 24.5% (FI≥0.3). Among those who fell, post-traumatic stress ranged from 3.4% (FI<0.1) to 36.9% (FI≥0.3). Falls were not significantly associated with post-traumatic stress among people who had an FI<0.3, but among those with an FI≥0.3 the odds ratio for having post-traumatic stress for those who fell was 2.25 (95% CI 1.2-4.23, p=0.011) compared to non-fallers. In conclusion, high levels of frailty can impact how a stressor, such as a fall, can be associated with an adverse psychological outcome.

